# Supplementary material for: An improved model for prediction of de novo designed proteins with diverse geometries
Source: bioRxiv. 2025 Jun 6:2025.06.02.657515. Preprint. [Version 1] doi: 10.1101/2025.06.02.657515 (PMC12157515; doi:10.1101/2025.06.02.657515)
Supplement: 1 [file NIHPP2025.06.02.657515v1-supplement-1.pdf]

bioRxiv preprint doi: https://doi.org/10.1101/2025.06.02.657515; this version posted June 6, 2025. The copyright holder for this preprint (which was not certified by peer review) is the author/funder, who has granted bioRxiv a license to display the preprint in perpetuity. It is made available under aCC-BY 4.0 International license.

SUPPLEMENTARY FIGURES

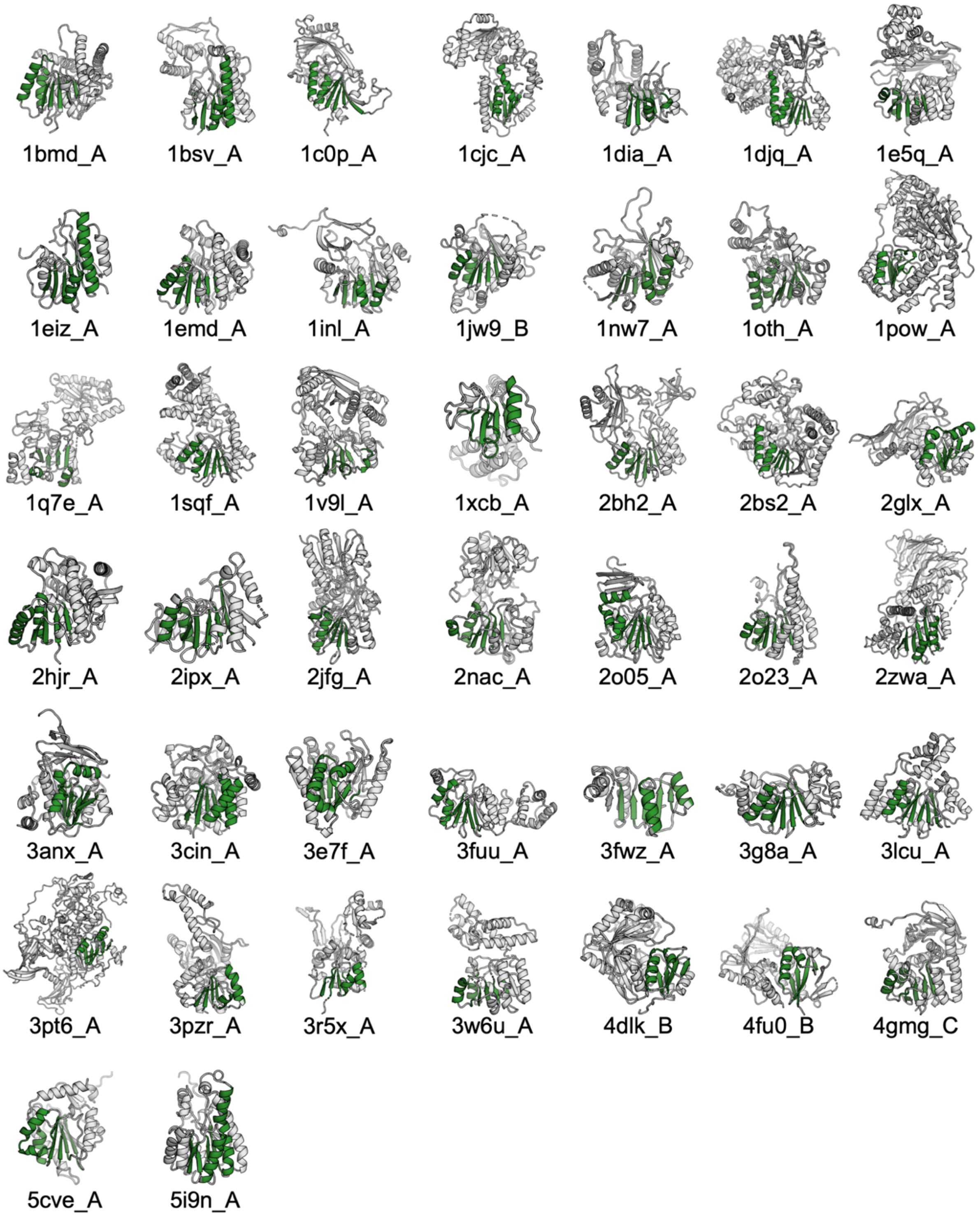

Supplementary Figure 1. 44 natural members of the Rossmann fold family. 44 natural members of the Rossmann fold family were taken from ref.[33]. Rossmann fold

bioRxiv preprint doi: https://doi.org/10.1101/2025.06.02.657515; this version posted June 6, 2025. The copyright holder for this preprint (which was not certified by peer review) is the author/funder, who has granted bioRxiv a license to display the preprint in perpetuity. It is made available under aCC-BY 4.0 International license.

beta strands and topology-matched helices to the two reshaped LHLs in 2LV8 are colored green.

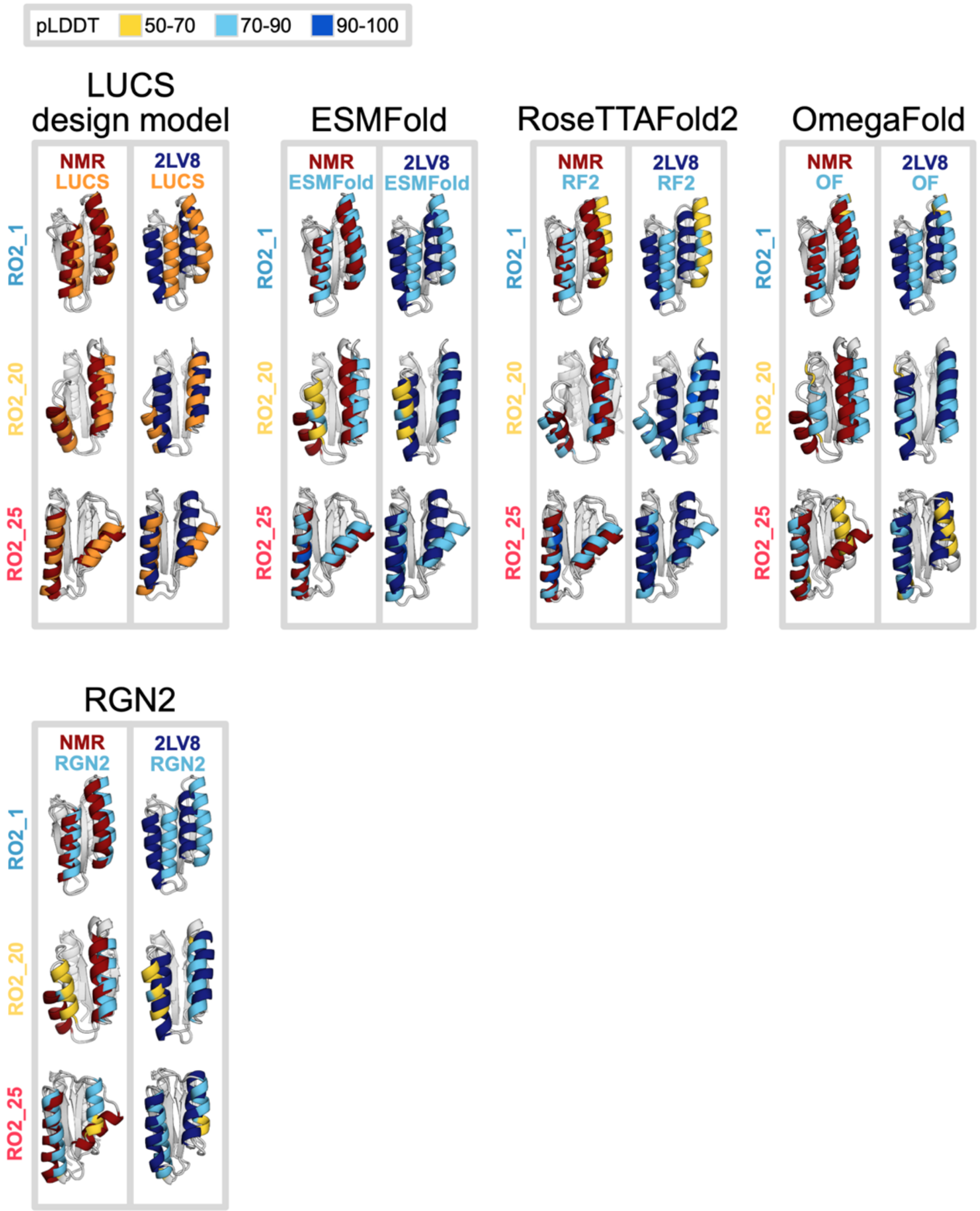

Supplementary Figure 2. Structure prediction for three LUCS designs with experimentally determined NMR structures using ESMFold, RoseTTAFold2 (RF2), OmegaFold (OF), and RGN2. The LUCS design models for these three designs are also shown. Predicted structures are compared to their lowest-energy NMR structures and 2LV8 (an idealized, de novo designed 2x2 Rossmann fold protein). NMR structures are shown in dark red, 2LV8 is shown in dark blue, the LUCS design model is shown in orange, and the predicted structures are colored by pLDDT for the given prediction method. AlphaFold3[2] (AF3) was excluded from this analysis as it currently does not support excluding template inputs of homologous protein structures. As the NMR

bioRxiv preprint doi: https://doi.org/10.1101/2025.06.02.657515; this version posted June 6, 2025. The copyright holder for this preprint (which was not certified by peer review) is the author/funder, who has granted bioRxiv a license to display the preprint in perpetuity. It is made available under aCC-BY 4.0 International license.

structures of the three LUCS designs are deposited in the PDB, AF3 is able to take the ground truth structures of these sequences as inputs during inference.

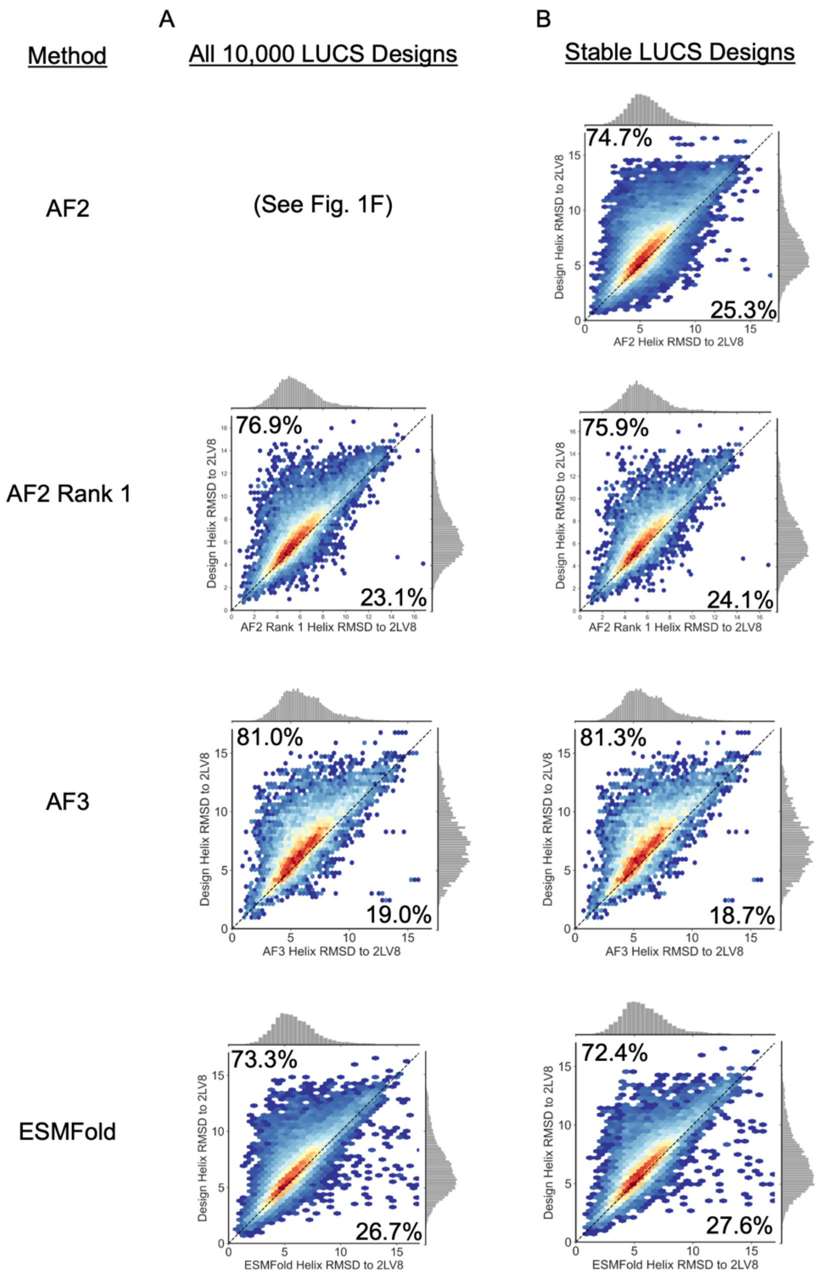

Supplementary Figure 3. Structure prediction of Rossmann fold LUCS designs using deep learning-based protein structure prediction methods. Shown are comparisons of RMSDs for the two reshaped LUCS helices to 2LV8 for the LUCS design model (y axis) and the predicted structure from each model (x axis) for: (**A**) The 10,000 experimentally tested Rossmann fold LUCS designs and (**B**) the 5,996 stable Rossmann fold LUCS designs. All the tested structure prediction methods show a

bioRxiv preprint doi: https://doi.org/10.1101/2025.06.02.657515; this version posted June 6, 2025. The copyright holder for this preprint (which was not certified by peer review) is the author/funder, who has granted bioRxiv a license to display the preprint in perpetuity. It is made available under aCC-BY 4.0 International license.

substantial bias towards the more idealized 2LV8 structure (density above diagonal, with percentage of designs indicated).

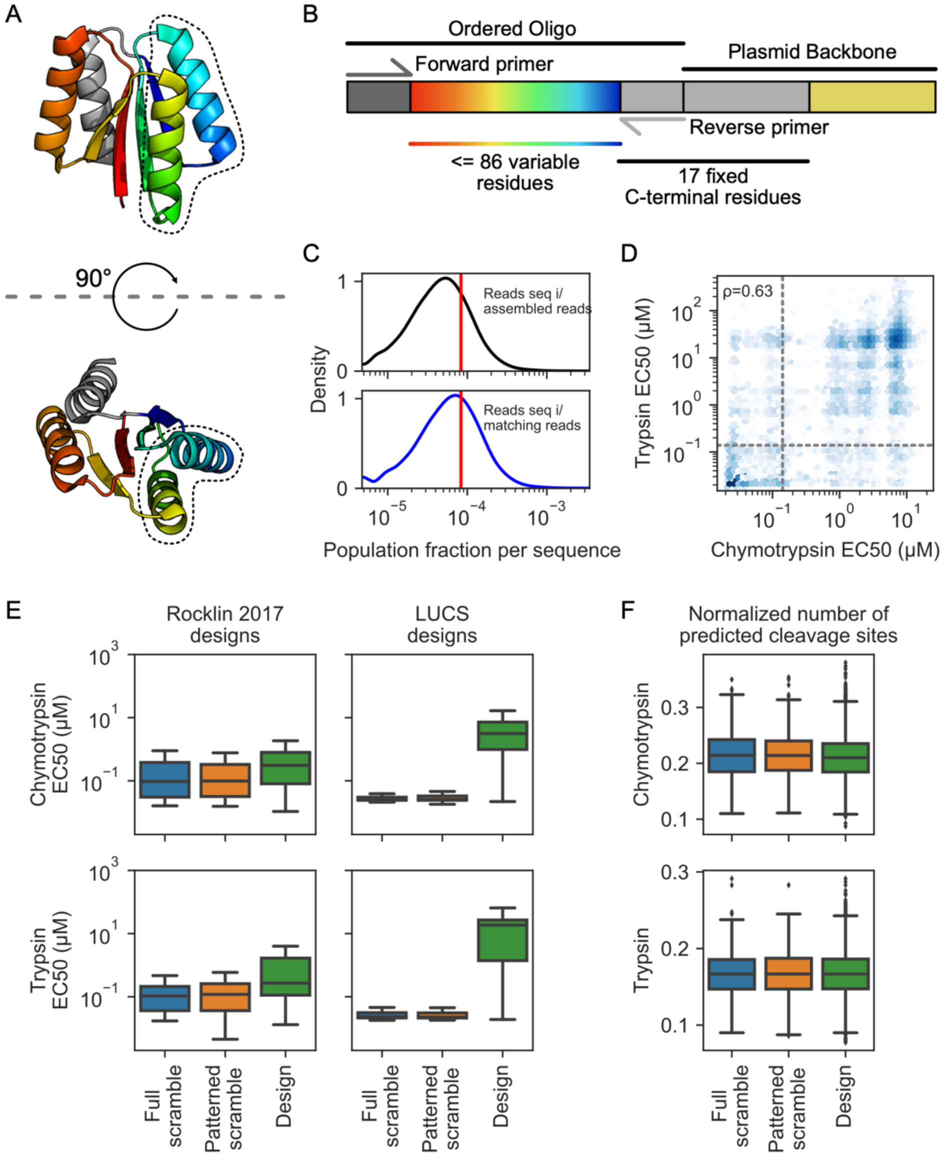

Supplementary Figure 4. Detailed analysis of yeast display. (**A**) De novo Rossmann fold (PDB: 2LV8) showing reshaped helices on the front side of the protein (black dotted outline) and residue positions redesigned during sequence design (rainbow, red to blue representing N-term to C-term). Residue identities kept fixed during sequence redesign due to oligo pool length restrictions (300 bp) shown in gray. (**B**) Oligos were ordered with 21 base pairs on the 5' (forward primer) and 3' (reverse primer) ends with homology to the destination vector for library amplification and cloning by yeast assembly. This left up to 258 variable nucleotides (which encode up to 86 variable residues, shown in rainbow). The reverse primer region encodes the first 7 amino acids of the 17 C-terminal residues that were kept fixed in all designs (gray), with the plasmid backbone encoding the remaining 10 fixed C-terminal residues, which enabled testing designs with up to

bioRxiv preprint doi: https://doi.org/10.1101/2025.06.02.657515; this version posted June 6, 2025. The copyright holder for this preprint (which was not certified by peer review) is the author/funder, who has granted bioRxiv a license to display the preprint in perpetuity. It is made available under aCC-BY 4.0 International license.

103 residues in total. (**C**) Number of reads corresponding to ordered designs in the naive library expressed as a population fraction of all assembled reads (black, top panel) or assembled reads matching ordered sequences (blue, bottom panel). The expected population fraction for the ordered library of 12,000 sequences shown as a red line. (**D**) Estimated chymotrypsin and trypsin EC50s for all sequences are modestly correlated by Spearman rho (ρ=0.63). Dotted lines represent the 95th percentile of patterned scramble sequences used as a cutoff for stable designs, such that stable designs are found in the upper right quadrant. (**E**) Estimated chymotrypsin and trypsin EC50s for all sequences tested here (LUCS designs) or previously designed de novo miniproteins[25]. Negative controls (fully scrambled sequences and patterned scramble sequences – scrambled sequences that maintain the original sequence's hydrophobic-polar pattern) show a clear separation from the designed sequences, with a much greater observed separation between controls and designs for LUCS designed proteins than de novo miniproteins. (**F**) Predicted number of chymotrypsin (top panel) and trypsin (bottom panel) cleavage sites across scramble controls and ordered designs by Rapid Peptides Generator[34] are similar between scramble controls and designs.

bioRxiv preprint doi: https://doi.org/10.1101/2025.06.02.657515; this version posted June 6, 2025. The copyright holder for this preprint (which was not certified by peer review) is the author/funder, who has granted bioRxiv a license to display the preprint in perpetuity. It is made available under aCC-BY 4.0 International license.

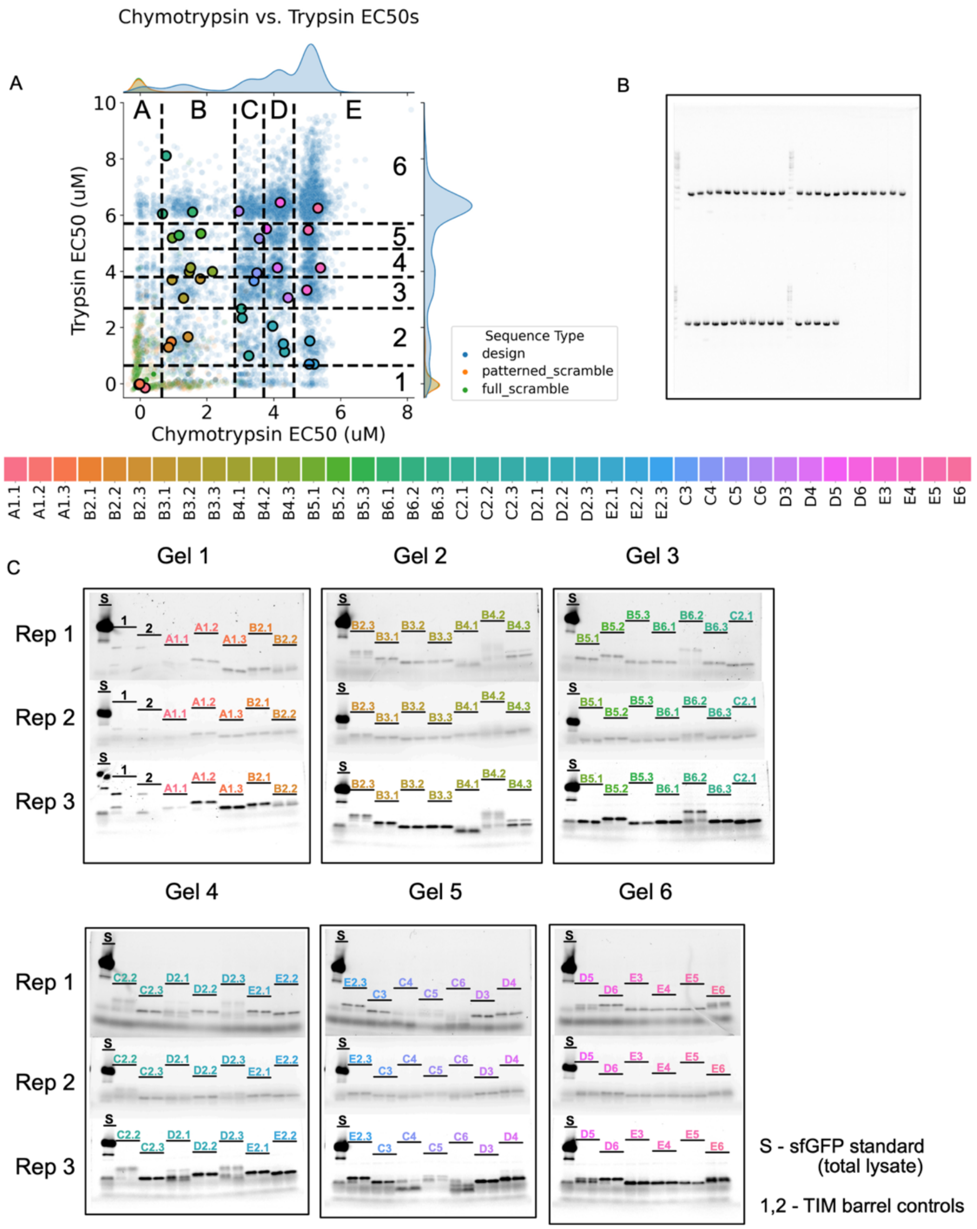

Supplementary Figure 5. Expression tests for 39 Rossmann fold LUCS designs. (**A**) 39 designs were sampled from chymotrypsin and trypsin EC50 bins. For each bin, the design with the median helix RMSD to 2LV8 was selected. For bins in which three designs were sampled, the designs at the 25th and 75th percentiles for helix RMSD to 2LV8 were also selected. (**B**) In-gel fluorescence shows successful PCR amplification for all 39 genes. (**C**) Protein expression and solubility were tested using a cell-free protein synthesis and solubility assay (Methods). Gels show fluorescence emission at 500 nm. Each pair of lanes for each sample contains the total lysate (left) and soluble fraction (right). The total lysate of a positive control gene, encoding Superfolder GFP (sfGFP)[35], was used as a standard for each gel. Controls 1 and 2 were de novo designed TIM barrels, which express but are not present in the soluble fraction.

bioRxiv preprint doi: https://doi.org/10.1101/2025.06.02.657515; this version posted June 6, 2025. The copyright holder for this preprint (which was not certified by peer review) is the author/funder, who has granted bioRxiv a license to display the preprint in perpetuity. It is made available under aCC-BY 4.0 International license.

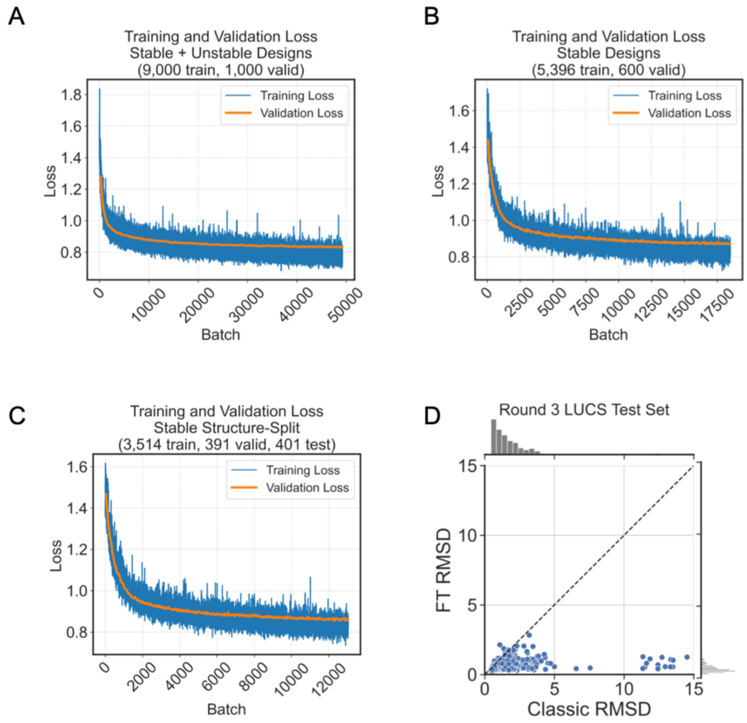

Supplementary Figure 6. Details for fine-tuning AF2 on LUCS design test set. Training (blue) and validation (orange) loss curves for three fine-tuned AF2 models: (**A**) Stable + Unstable, (**B**) Stable, and (**C**) Stable Structure-Split. (**D**) Performance of Stable Structure-Split FT-AF2 on its test set, generated using a structure-based split in which no test set example had a helix RMSD of 2 Å or less to any topology-matched reshaped helix in the training or validation set (Methods). Stable Structure-Split greatly outperforms classic AF2 in predicting these test set examples, which likely indicates that this test set is an easy test set given the training data.

bioRxiv preprint doi: https://doi.org/10.1101/2025.06.02.657515; this version posted June 6, 2025. The copyright holder for this preprint (which was not certified by peer review) is the author/funder, who has granted bioRxiv a license to display the preprint in perpetuity. It is made available under aCC-BY 4.0 International license.

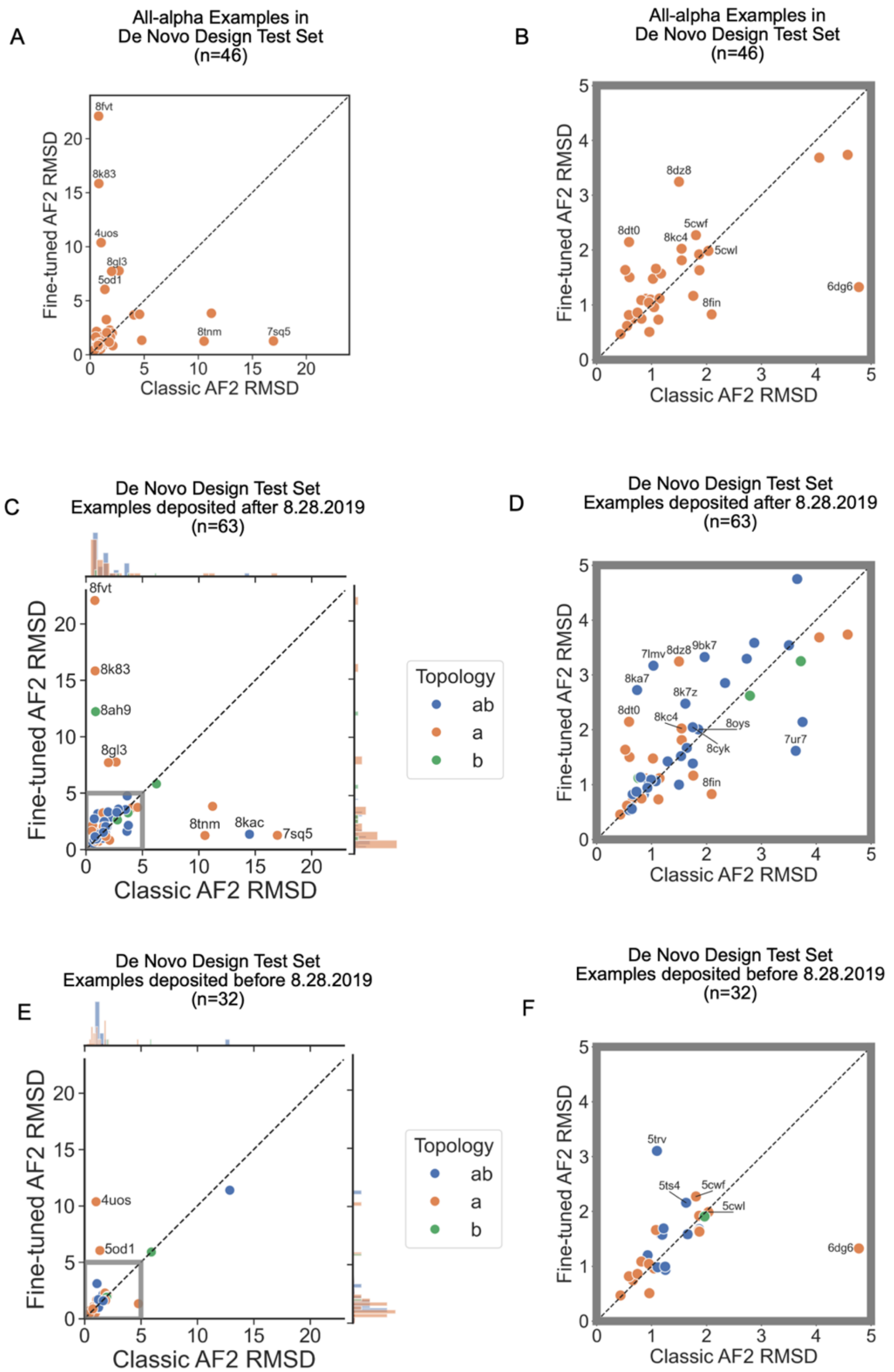

Supplementary Figure 7. Details of predictions for de novo test set. (A) Predictions for the 46 all-alpha proteins in the de novo protein test set by Classic and Stable Structure-Split FT-AF2. Axes limits trimmed to 5 Å in (**B**). (**C**) De novo test set filtered by PDB deposition date after the AF2 training set cutoff (August 28th, 2019). Axes limits trimmed to 5 Å in (**D**). (**E**) De novo test set filtered by PDB deposition date before the AF2 training set cutoff (August 28th, 2019). Axes limits trimmed to 5 Å in (**F**). Examples predicted to < 2 Å RMSD to the ground truth by one model and not the other are labeled with their PDB IDs.
